# Rapid Acetabular Chondrolysis following Hemiarthroplasty of the Hip: A Poor Prognostic Sign

**DOI:** 10.1155/2019/7328526

**Published:** 2019-05-07

**Authors:** Abidemi Adenikinju, James D. Slover, Kenneth A. Egol

**Affiliations:** NYU Langone Orthopedic Hospital, NYU Langone Medical Center, New York, NY, USA

## Abstract

Both hemiarthroplasty and total hip arthroplasty have been well described as effective methods of management for displaced femoral neck fractures in the elderly. Acetabular erosion is a common long-term complication of hemiarthroplasty. We present a case in which rapid acetabular erosion occurs within weeks of hemiarthroplasty, ultimately leading to an acetabular fracture and need for revision to total hip arthroplasty. Early and rapid acetabular erosion following hip hemiarthroplasty has not been well documented in current literature. It may lead to acetabular fracture and may be secondary to infectious causes. If encountered, an infection workup should be initiated.

## 1. Introduction

Hemiarthroplasty and total hip arthroplasty have both been well described as effective methods of management for displaced femoral neck fractures in the elderly [1–5]. Total hip arthroplasty has superior functional outcomes and lower long-term revision rates [6–9], while hemiarthroplasty is associated with lower dislocation rates and faster operative times. While hemiarthroplasty remains an appropriate management option in low-demand patients, it is commonly complicated by acetabular erosion [3, 7, 10, 11].

Acetabular erosion has a reported incidence of up to 66% [10–12]. It is typically a late complication, taking place months to years after the surgery [8, 13–17], and can result in severe pain, which impedes functional outcomes and may ultimately lead to revision surgery [8, 10–12, 14, 15, 18, 19]. The etiology is unclear but may include direct injury from initial trauma as well as wear of the native cartilage against a nonanatomic bearing surface. It has been correlated with increased activity and length of time from surgery [15]. Although generally thought of as a chronic process [13–15, 20, 21], we present a case in which acetabular erosion occurs rapidly, within two weeks of a hemiarthroplasty in the treatment of a femoral neck fracture, ultimately leading to an acetabular fracture and need for revision to total hip arthroplasty.

## 2. Case Report

**Figure 1:**
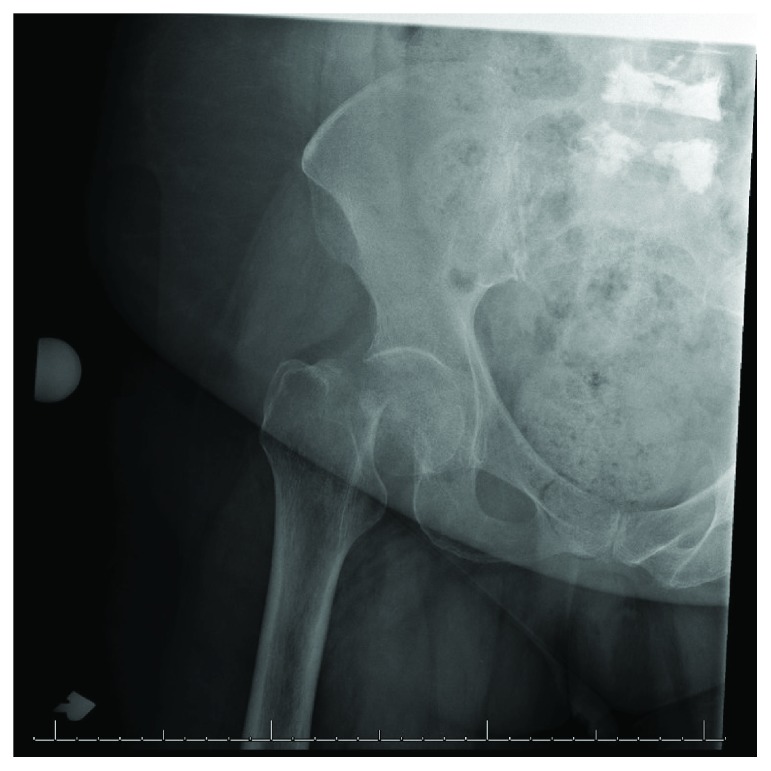
AP radiograph of the right hip demonstrating a displaced femoral neck fracture.

**Figure 2:**
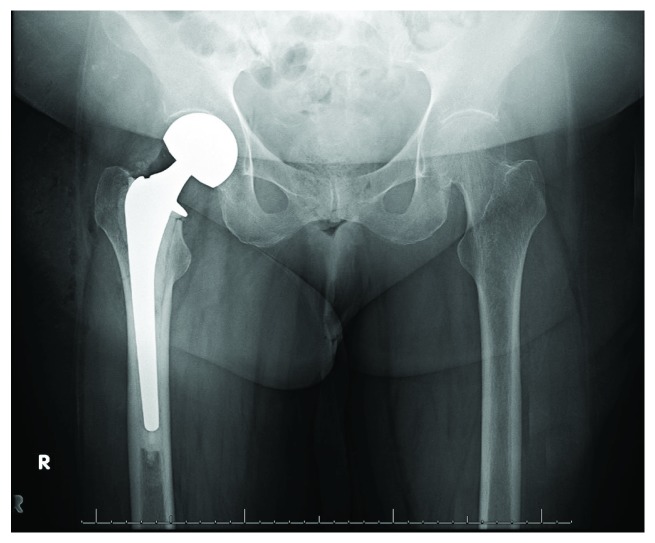
AP pelvis radiograph immediately postop following right hip bipolar hemiarthroplasty.

A 73-year-old woman presented with right hip pain and inability to ambulate after a mechanical fall in her home. The patient had a past medical history of congestive heart failure, atrial fibrillation, and hyperlipidemia. Three months prior to the fall, the patient had been hospitalized for 10 days due to a polymicrobial (Pseudomonas aeruginosa, Proteus vulgaris, non-beta-hemolytic streptococci, and Escherichia coli) urinary tract infection, was subsequently treated at a rehabilitation facility for a 6-week period, and was ultimately discharged home. Two days following discharge, she sustained a mechanical fall and presented to the emergency department. Prior to her fall, she was minimally ambulatory with a walker due to deconditioning from the recent hospitalization as well as a remote history of bilateral Achilles tendon injuries resulting in equinus contractures. The patient denied antecedent hip pain or pain in other joints on presentation. On physical exam, she was noted to be obese with a BMI of 36.56, her right lower extremity was shortened and externally rotated, and she had diffuse tenderness about the hip. She was neurovascularly intact distally. Radiographs including an anteroposterior (AP) pelvis and AP and lateral view of the hip demonstrated a displaced femoral neck fracture (Figure 1). Due to medical comorbidities and limited functional status prior to the injury, the plan was made to proceed with a hemiarthroplasty as opposed to total hip arthroplasty. Two days following admission, the patient underwent a cemented hemiarthroplasty via the posterior approach to the hip without complication (Figure 2). During her hospitalization, complete blood count and basic metabolic panel were monitored and within normal limits by discharge. She was diagnosed with osteoporosis on admission (left femoral neck *T* score of -2.8 on dual-energy X-ray absorptiometry [DEXA] scan). Her PTH level was slightly elevated (97.8 pg/mL), but both calcium and vitamin D levels were within normal limits (9.2 mg/dL and 41.8 ng/mL, respectively). An immunofixation test to detect multiple myeloma was unremarkable. She was ultimately discharged to a skilled nursing facility on postoperative day 4 with prescriptions for low molecular weight heparin for deep vein thrombosis (DVT) prophylaxis, narcotics for pain control, and vitamin D. She was also instructed to follow up with a metabolic bone disease specialist for further management of osteoporosis.

**Figure 3:**
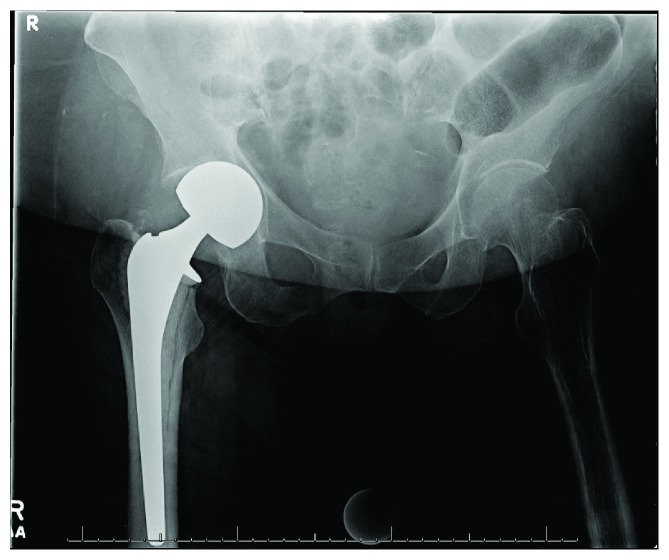
AP pelvis radiograph obtained 2 weeks postop demonstrating joint space narrowing and medialization of femoral component.

Two weeks after discharge, she presented with right posterior thigh and groin pain, which she reported had been constant since surgery and had prevented progress with physical therapy. She denied a new traumatic event. Physical exam was notable for tenderness over the proximal hamstring but was otherwise normal. Radiographs (Figure 3) and computed tomography (CT) scan demonstrated increased medialization of the femoral component into the acetabulum without evidence of loosening or hardware complications. Bilateral lower extremity duplex was negative for DVT. Infection workup revealed CRP 6 mg/L, WBC 11 (10^3^/*μ*L), and ESR 75 mm/hr, and it was determined that she did not have a periprosthetic joint infection (PJI) at that time. She was admitted overnight for pain management and discharged back to the skilled nursing facility the next day with a plan for restricted weight bearing with a walker.

**Figure 4:**
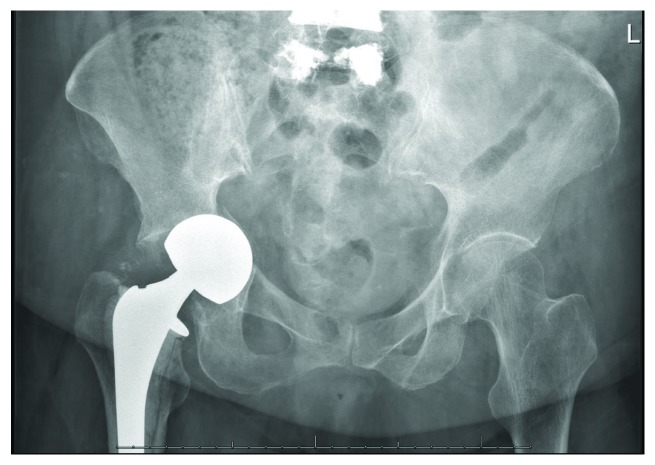
AP pelvis radiograph at 6 weeks postop demonstrating fracture at the medial acetabular wall with superomedial migration of the femoral head, protrusio acetabuli, and no evidence of femoral component loosening.

**Figure 5:**
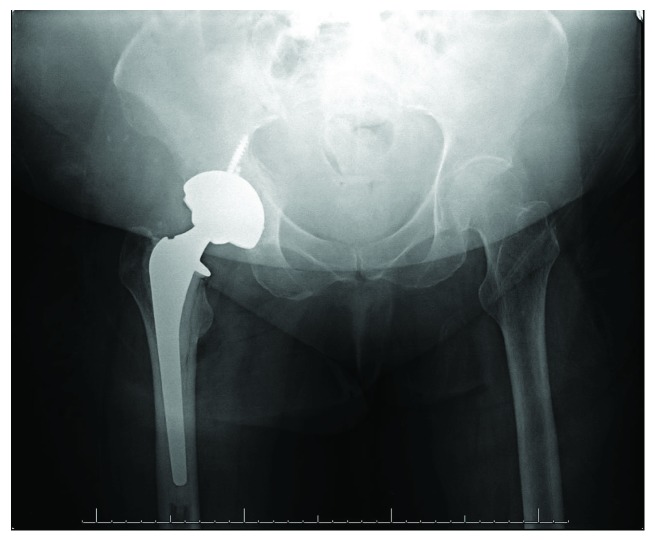
AP pelvis radiograph obtained immediately following conversion to right total hip arthroplasty.

At the 6-week postoperative visit, she continued to have limited progress with physical therapy, which she attributed to ongoing groin pain and weakness. Exam was within normal limits. Radiographs (Figure 4) and CT again demonstrated increased medialization of the femoral head, now with a fracture of the medial wall of the acetabulum. Infection workup revealed CRP < 5 mg/L, WBC 9.7 (10^3^/*μ*L), and ESR 71 mm/hr, and it was again determined that she did not have PJI at that time. She underwent conversion to a right total hip arthroplasty the next day (Figure 5) via a posterior approach. The fracture was treated with a jumbo cup and screws with impaction grafting technique utilized to restore medial bone loss. There were no complications. She was made partial weight bearing with instructions for walker-assisted ambulation at all times and discharged on postoperative day 4.

**Figure 6:**
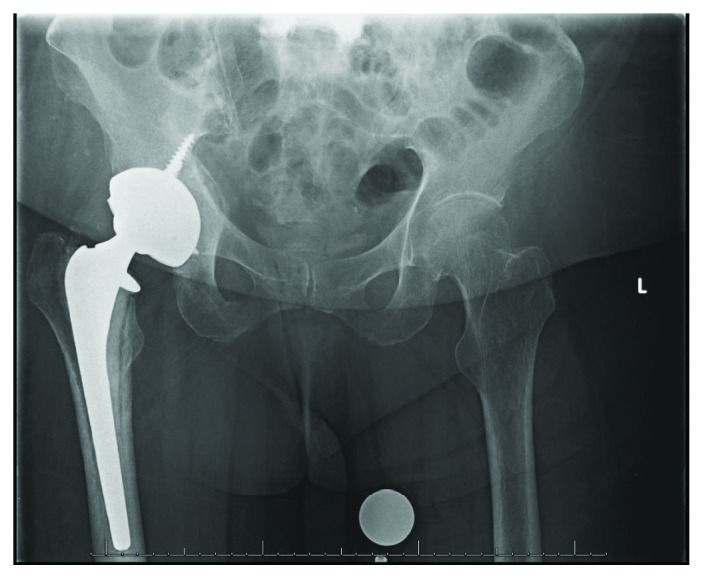
AP pelvis radiograph obtained 2 weeks after revision to total hip arthroplasty. No evidence of component loosening. Fracture line without displacement.

**Figure 7:**
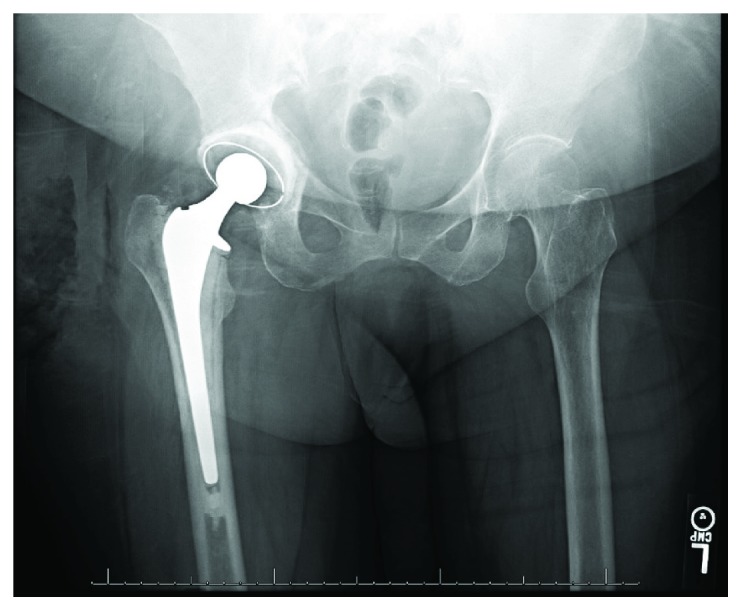
AP pelvis radiograph obtained following right revision total hip arthroplasty.

Two weeks after discharge, she presented with a 1-day history of incisional drainage. Her exam was significant for a fever of 101°F and an erythematous indurated incision with serous drainage. Infection workup revealed WBC 13.9 (10^3^/*μ*L), ESR 110 mm/hr, and CRP 89 mg/L. Radiographs did not demonstrate evidence of component loosening or fracture displacement (Figure 6). She underwent right hip irrigation and debridement and revision arthroplasty the next day (Figure 7). A cemented all-polyethylene cup was used in order to remove the bone graft and allow for the use of antibiotic cement. Intraoperative cultures grew Enterobacter cloacae, which was not one of the organisms of her previous UTI. The infection was treated with vancomycin and cefepime with a planned 6-week course as recommended by infectious disease, given that gram-positive involvement had not been entirely ruled out. She maintained partial weight bearing status and was discharged to subacute rehab on postoperative day 3.

**Figure 8:**
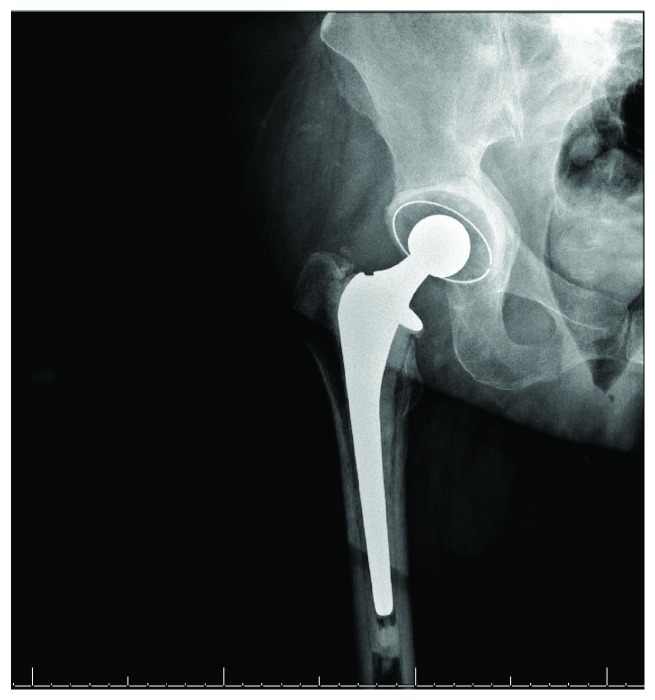
AP radiograph right hip obtained 3 weeks after revision total hip arthroplasty demonstrating no evidence of component loosening.

She reported to the outpatient clinic for follow-up 3 weeks later. She continued to progress with physical therapy. Physical exam and imaging (Figure 8) were normal, and she was transitioned to weight bearing as tolerated.

## 3. Discussion

Erosion of acetabular bone and cartilage is a common late complication of hemiarthroplasty, which can result in pain and component migration, with eventual need for revision as seen in this case [12–14, 16, 17]. The etiology of acetabular erosion is often multifactorial.

One of the most common mechanisms is wear [22–26]. The shedding of metal, polyethylene, or cement particles leads to an inflammatory reaction that can cause both osteolysis and cartilage degeneration [16, 26]. Though the bipolar hemiarthroplasty design has more opportunity for motion producing wear given the metal-polyethylene interface, the design also theoretically results in less stress on the acetabular surface, which would theoretically lead to less acetabular erosion [11, 16, 25, 26]. This could explain why studies have shown higher incidence of acetabular erosion with unipolar prostheses [11, 17]. These processes, however, are gradual and do not result in radiographic findings or symptoms for months to years.

PJI is a known cause of osteolysis and has been reported as a predisposing factor of protrusio acetabuli [26–28]. PJI leads to an inflammatory cycle, consisting of cytokine release, proteolytic enzyme activation, and upregulation of osteoclasts, resulting in rapid and aggressive destruction of bone and cartilage [26, 28–30]. Lim et al. report chronic infection as a predisposing factor of component migration and protrusion after hemiarthroplasty, but even in that setting, it occurred 5 years after the index surgery [28]. Despite the fact that the organism identified from the intraoperative cultures of the last revision was not one of the bacteria associated with the patient's previous UTI, it remains possible that an indolent infection not only played a role but also was the main etiology of the rapid chondrolysis seen in this case.

Patient factors may have also influenced the outcome of this case. Though it has not been well-defined in literature, osteoporosis possibly plays a role in long-term acetabular chondrolysis. Finnilä et al. measure the migration of acetabular components in total hip arthroplasty and find that patients with low bone mineral density continue to have significant cup migration for up to twelve months, well past the initial three-month settling period [20]. Hedbeck et al. report that acetabular erosion was more common in patients with low body mass index (BMI), which was unusual given that obesity had previously been identified as a predisposing factor for acetabular erosion in a study by Davalillo et al. [12, 31]. Presumably, increased body weight leads to increased wear on the acetabulum causing more acetabular erosion. Hedbeck et al. hypothesized that osteoporosis likely contributed to their finding, given that osteoporosis is more common in patients with low body weight [12]. In this case, the combination of osteoporosis and obesity may have contributed to the rapid degeneration that occurred.

There are no cases in current literature describing acetabular erosion occurring within the first few weeks of the postoperative period. Sen et al. describe a case in which acetabular erosion occurred 5-6 months following a primary hip hemiarthroplasty, but the etiology of the early onset was unclear [15]. Osteolysis occurring within months of arthroplasty has been described in case reports as a manifestation of pathologic processes including Paget's disease, malignancy, and vascular malformation [24, 32–34]. Though rare, it is possible that these disease processes could also contribute to cartilage degeneration and should be included on the differential. An unrecognized fracture or subchondral insufficiency fracture is also possible, though there was no evidence of acetabular fracture on initial perioperative imaging or intraoperative observation in this case.

In conclusion, acetabular erosion occurs commonly as a late complication of hemiarthroplasty. Though not well documented in current literature, early and rapid development of this complication may lead to acetabular fracture. The etiology of acetabular erosion is multifactorial, with infection, osteoporosis, and obesity likely serving as key contributing factors. If encountered, an infection workup should be initiated. It is unknown whether early treatment of periprosthetic hip infection, if present, would have interrupted the natural course in this case or whether the ultimate outcome could have been altered.
